# A predictive model of offspring congenital heart disease based on maternal risk factors during pregnancy: a hospital based case-control study in Nanchong City

**DOI:** 10.7150/ijms.48046

**Published:** 2020-10-22

**Authors:** Yun Liang, Xiaoqin Li, Xingsheng Hu, Bing Wen, Liang Wang, Cheng Wang

**Affiliations:** 1 Department of Pediatric Surgery, Affiliated Hospital of North Sichuan Medical College, Nanchong, 637000, P.R. China; The first affiliated Hospital of Jinan University, Guangzhou, 510632, P.R. China.; 2 Department of Nursing, Affiliated Hospital of North Sichuan Medical College, Nanchong, 637000, P.R. China.; 3 Department of Cardiology, Nanchong Central Hospital, Nanchong, 637000, P.R. China; Department of Oncology, the second Xiangya Hospital of Central South University Changsha, Yuelu District, 410011, P.R. China (Current Address).; 4 Department of Cardiothoracic Surgery, Nanchong Central Hospital, Nanchong, 637000, P.R. China.

**Keywords:** congenital heart disease, risk factors, case-control matching, risk model

## Abstract

**Objective:** Based on epidemiological field data, this study was to develop a prediction model which can be used as a preliminary screening tool to identify pregnant women who were at high risk of offspring congenital heart disease (CHD) in Nanchong City, and be beneficial in guiding prenatal management and prevention.

**Methods:** A total of 367 children with CHD and 367 children without congenital malformations aged 0 to 14 years old were recruited from the Affiliated Hospital of North Sichuan Medical College and Nanchong Central Hospital between March 2016 and November 2018. Using the SPSS 22.0 case-control matching module, the controls were matched to the cases at a rate of 1:1, according to the same gestational age of child (premature delivery or full-term), the maternal age of pregnancy (less than 1 year). 327 matched case-control pairs were analyzed by SPSS 22. Univariate and multivariate analysis were performed to find the important maternal influencing factors of offspring CHD. A logistic regression disease prediction model was constructed as the final predictors, and Hosmer-Lemeshow goodness of fit test and receiver operating characteristic (ROC) curve were used to evaluate the model.

**Results:** 654 subjects (327 cases and 327 controls) were matched. The 25 variables were analysed. The logistic regression model established in this study was as follows: Logit(P)= -2.871+(0.686×respiratory infections)+(1.176×water pollution)+(1.019×adverse emotions during pregnancy) - (0.617×nutrition supplementation). The Hosmer-Lemeshow chi-square value was 7.208 (df = 6), with a nonsignificant p value of 0.302, which indicates that the model was well-fitted. The calibration plot showed good agreement between the bias-corrected prediction and the ideal reference line. Area under the ROC curve was 0.72 (95% CI: 0.681~0.759), which means that the predictive power of the model set fitted the data.

**Conclusion:** In Nanchong city, more attention should be paid to mother who had a history of respiratory infections, exposure to polluted water, adverse emotions during pregnancy and nutritional deficiency. The risk model might be an effective tool for predicting of the risk of CHD in offspring by maternal experience during pregnancy, which can be used for clinical practise in Nanchong area.

## Introduction

Congenital heart disease (CHD) is the most common type of structural congenital malformation and the primary non-infectious cause of death in children: the estimated worldwide prevalence of CHD is about 4-10 in 1,000 live births or 19 in 1,000 live births if potentially serious cases of bicuspid aortic valve are included 1. In China, the prevalence is 7 to 8 per 1000 live births in high-prevalence areas, which represent over 100 000 new cases of CHD per year 2. Several studies have demonstrated an association between environmental risk factors and fetus CHD, and the maternal environment in which a fetus develops has a profound effect on its developmental trajectory, impacting fetal cardiac structure development 3. Substantial epidemiological evidence associates the sustained presence of adverse healthy lifestyle factors with increased risk of CHD mortality 4. However, few studies have been done to predict the risk of individual congenital malformations, including CHD 5.

Nanchong, which lies in the northeastern of the Sichuan Basin, is not just a populous city in China, but also an agriculture-oriented developing city 6. With the resident relocation, urbanization and economic development, pollutants from industrial and domestic wastewaters and agricultural runoffs had exceptionally affected the ecological environment in the Nanchong region 7. Because of the need of urbanization construction, migrant workers from impoverished rural areas moved to flourishing cities for seeking better job opportunities and pursuing dreams of a better life 8. These migrant workers, the young in particular, faced many challenges, such as economic pressure, work load, family separation, discrepancies between expectations and reality, and acculturative stress 9. In order to save money, these people didn't wish to go to the hospital for health examination regularly, and they did not pay enough attention to the health of their offspring 10.

Previously, by using univariate and multivariate methods, we explored the possible parental nonhereditary exposure factors relevant to the occurrence of CHD in the northeastern Sichuan area and evaluated the relative importance of each risk factor 11. However, we didn't develop an effective CHD prediction model built on comprehensive epidemiological data.

The aim of this study was to establish a prediction model which can be used as a preliminary screening tool to identify pregnant women who were at high risk of offspring CHD in Nanchong City, and be beneficial in guiding prenatal management and prevention.

## Materials and methods

### Subjects

In this hospital-based case-control study, subjects were recruited in two tertiary hospitals from the Affiliated Hospital of North Sichuan Medical College and Nanchong Central Hospital in Nanchong between March 2016 and November 2018. Mothers who gave birth to CHD infants were involved as cases. CHD was diagnosed by echocardiography. The control group consisted of the mothers whose children were undergoing physical disease treatment (for example, upper respiratory tract infection) in the same period. The exclusion criterions were: chromosome abnormality (for example, Downs syndrome) and other inherited familial diseases; refusal or inability to participate in the survey because of mental symptoms, thinking, or memory disorders.

In this study, to diminish potential risk of bias as far as possible and ensure the statistical power needed to perceive an important predictor, we carried out a case-control study with 1:1 frequency matching.

Informed consent was obtained from all individuals before the interview. The ethical approval of this project was authorized by the Affiliated Hospital of North Sichuan Medical College Ethics Committee [No. 2015ER(A)030]. All the procedures in this study conformed to the Declaration of Helsinki.

### Data collection

All subjects were interviewed face-to-face by well-trained cardiologist and cardiac surgeon and called upon to fill in a questionnaire. The questionnaire included: sociodemographic characteristics, pregnancy history, family history, environmental risk factors, and lifestyle behaviour during pregnancy. The questionnaire was designed by the experts from our research team and modified based on research literature reported by other scholars.

### Measurements of risk factors

#### Sociodemographic characteristics

Ethnicity was classified into 2 categories: Han and minorities (minorities were the other 55 ethnicities in China except Han) 12. Residence was divided into downtown and rural area 13. Education level was classified into 3 categories: primary school and below; middle school; college and above 2. Body Mass Index (BMI) was thin (≤18.4), normal (18.5-23.9), overweight (24.0-27.9) and obese (≥28.0) 14.

#### Pregnancy history

Maternal pregnancy history consisted of history of abnormal reproduction (stillbirth, spontaneous abortion, or birth defect) 2, respiratory infections (for example, pneumonia pharyngitis, influenza or “colds”) 15, and medication history (more than 1 day of taking any drug that was used to treat a disease) 16.

#### Family history

Family history of CHD was defined as 1 or more first relatives of a CHD patient 15.

#### Environmental risk factors

Information about exposure to environmental risk factors was collected using the questions with the answer “yes or no”. The exposure time of maternal risks was defined as from “6 months before conception” to “the first trimester of pregnancy” daily. Information about exposure to environmental risk factors included electric radiation (daily contact with mobile phones, microwave ovens, induction cookers, and computers for more than 30 minutes), heavy metals, pesticides (insecticides, herbicides, rodenticide, etc.), organic poisons (lead, mercury, cadmium, benzene, paint, hair dye and other recognized harmful chemical agents), noise (working or living in a noisy environment that causes maternal discomfort), air pollution(referred to the existence of exhaust gas near the plant, a distance from the main traffic road of less than 50 mile or work in the coal-fired power plant), air pollution (the air quality was determined by the local environmental protection department to be at the pollution level), and water pollution (defined as the pollution of drinking water or irrigation water for crops near the factory where the waste water was discharged or recognized by the local environmental protection department) 17.

#### Lifestyle behaviours

Maternal lifestyle behaviour was summarized from the same periods as environmental risk factors. Smoking was defined as smoking any cigarette during pregnancy 18. Alcohol drinking was defined as drinking any liquor, including beer, wine, and white spirits 2. Addictive drug taking was defined as taking any drugs, including heroin, cannabis, morphine, cocaine and ketamine 19. Sleep disorder referred to difficulty in falling asleep, light sleep, ease to arousal or waking up early 20. Adverse emotions during pregnancy referred to bad mood at least once a month, such as tension, injury, anxiety and depression, etc. 21.

### Statistical analysis

Basically there are four data analysis steps. In the first stage, using the SPSS 22.0 case-control matching module, the controls were matched to the cases at a rate of 1:1, according to the same gestational age of child (premature delivery or full-term), the maternal age of pregnancy (less than 1 year). In the second stage, univariate and multivariate analysis were performed to find the important maternal influencing factors of offspring CHD. In the third stage, a logistic regression disease prediction model was constructed as the final predictors. Finally, the predictive ability of the model was evaluated by receiver to operating characteristic curve (ROC) and Hosmer-Lemeshow test.

## Results

Initially, 734 subjects (367 cases and 367 controls) were enrolled. The difference between the gestational age of child and maternal gestational age may affect the duration of the foetus under the influence of non-genetic exposure factors, and were treated as potential confounders. We did a matched case-control study to identify risk factors. Finally, 654 subjects (327 cases and 327 controls) were matched (**Tables 1 & 2**).

**Figure 1:**
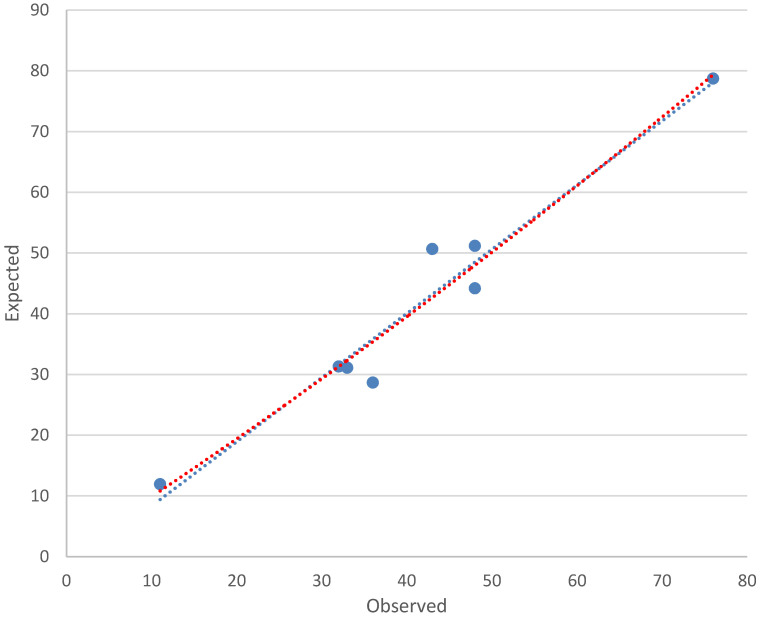
Scatter Diagram of Calibration.

**Figure 2:**
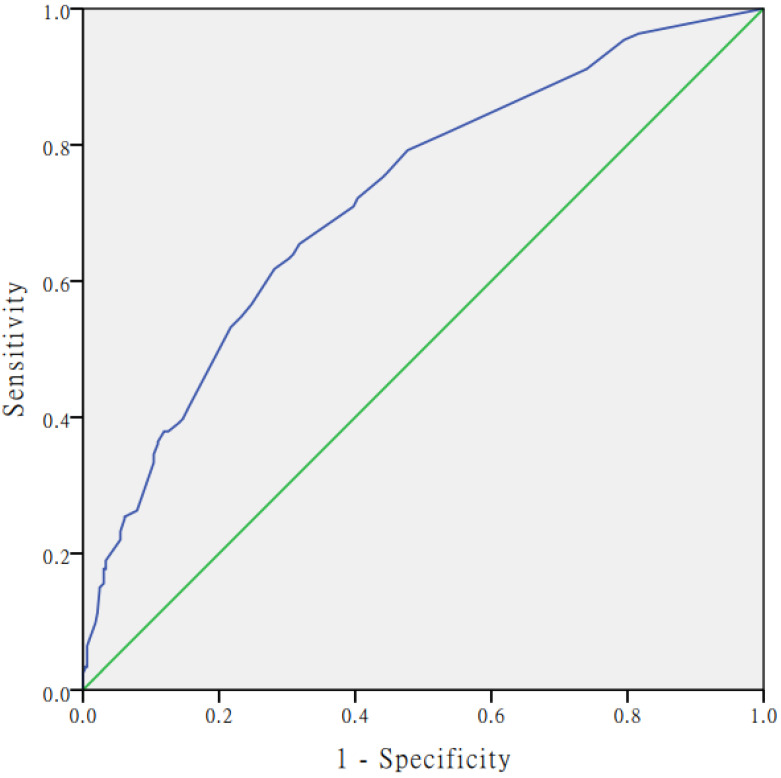
The receiver operating characteristic (ROC) curve of the multivariate logistic regression model.

The 25 variables listed in **Table 3** were analysed by univariate logistic regression. **Table 3** showed the 17 significant predictors of CHD risk selected by univariate logistic regression. The following 3 factors were significantly associated with the increased risk of CHD by multivariate logistic regression analyses (**Table 4**): respiratory infections **(**Β = 0.686, OR = 1.986, 95% CI: 1.336~2.952), exposure to polluted water (Β = 1.176, OR = 3.242, 95% CI: 1.872~5.613), adverse emotions during pregnancy (Β = 1.019, OR = 2.769, 95% CI: 2.005~3.824). The occurrence of CHD was inversely related to nutrition supplementation (Β = -0.617, OR = 0.540, 95% CI: 0.381~0.765) as a protective factors (**Table 4**). The logistic regression model established in this study was as follows: Logit(P)=-2.871+(0.686×respiratory infections)+(1.176 × water pollution)+(1.019×adverse emotions during pregnancy) - (0.617×nutrition supplementation). The Hosmer-Lemeshow chi-square value was 7.208 (df = 6), with a nonsignificant *p* value of 0.302, which indicates that the model was well-fitted. The calibration plot showed good agreement between the bias-corrected prediction and the ideal reference line (**Figure 1**). **Figure 2** shows the Receiver Operating Curve (ROC Curve). Area under the ROC curve was 0.72 (95% CI: 0.681~0.759), which means that the predictive power of the model set fitted the data.

## Discussion

The outcome of this study has shed more light on risk factors of CHD in offspring caused by maternal non-genetic exposure. This study has attempted to control for the effects of potential confounders by comprising many factors into the analyses without over-modelling. Effective nutrition supplementation was found to have an inverse relationship with offspring CHD. Inadequate maternal nutrition was a recognized risk factor in child development, which had been confirmed by many scholars 22. Prior studies showed that multivitamin or folic acid use before or during early pregnancy may antagonistically affect the biological process of vascular disruption and/or apoptosis, and seems to rescue apoptotic cells of folate deficient 23. Miki emphasized the significant interactions between fatty acid metabolism and further underscored the importance of proper maternal nutrition during gestational age for proper organ development and function among all vertebrate species 24. The results of this study emphasized again how important it is to get effective nutrition supplementation for mother.

In our study, maternal exposure to polluted water during pregnancy was risk factor for offspring CHD. With the rapid development of economy, water pollution had grown up to be a serious problem in China 25. At least 70 percent of lakes and rivers in China were polluted, with more than half of the water sources were unsuitable for human contact 26. Around 60% of China's underground water had inferior water quality and over 80% of petrochemical and chemical facilities were situated in easy reach of rivers and water tables 27. To date, more than 350 million people in the country had no access to safe drinking water, despite recent progress about improving the water quality of Chinese natural water sources 28. The reason for the above phenomena can be attributed to expanding construction of cities, over-used fertilizers and toxic chemicals and emissions from industries 29. Kim J et al reported that cardiac malformations were positively correlated with pollutants in drinking water, such as trichloroethylene, tetrachloroethylene, dichloromethane, and benzene 30. Meanwhile, it was confirmed that the risk of ventricular septal defect (VSD) can be increased by trichloroacetic acid, trihalomethane and dichloroethylene in drinking water 2,31. In Nanchong, there were two main sources of nitrate that pollute groundwater 32. One was the discharge of sewage and waste water from the surface, such as sewage from urban septic tanks, sewage leaked from sewage pipes, or garbage piles leached by rainwater 11. In the process of discharge, the polluted water from this source would seep into the river, thus polluting the groundwater resources 32. Secondly, agricultural non-point sources pollute the water source, which leaded to the over standard of nitrate and heavy metals in groundwater resources 32,33. It was usually necessary to apply nitrogen fertilizer in the farmland, so nitrogen will seep into the farmland; the content will be 12.5%-45% nitrogen fertilizer 34. When crops such as vegetables were irrigated with wastewater contaminated with heavy metals, dietary intake would become the main route of heavy metal exposure 33. Over the past few years, ecological environment of Nanchong city had been subjected to heavy metal accumulation as a result of rapid economic development, urbanization and industrialization 35,36. As mentioned before, environmental exposure factors were related to environmental pollution. Inappropriate urbanization and extensive economic growth had significant influences on the regional environment in the context of rapid urbanization in China 37. Thus, it can be considered as a warning that it is undesirable to develop the economy at the expense of the environment.

Our findings support those of other studies that maternal adverse emotions, a common morbidity during pregnancy, can lead to poor birth outcomes 38. Xiaoqiang, Q et al reported that psychological trauma or tension, stimulating the sympathetic adrenomedullin system and pituitary adrenocortical system, will cause a series of physiological changes and increase the risk of teratogenesis 39. Shaw, C et al reported that stressful events during pregnancy will lead to cardiac malformations of the outflow tract, neural tube malformations and cleft lip 40. Therefore, to maintain a positive mood may not only be beneficial for reproductive health but also for maternal health.

Evidence from the previous literature shows that prenatal maternal influenza during 3 months before pregnancy through the third month of pregnancy may be associated with right-sided obstructive lesions in all infants and with atrioventricular septal defects in infants with Down syndrome 41. Another study indicated that a febrile illness around the time of conception or in early pregnancy was associated with an approximately two-fold increased risk for major heart defects 42. The new data that reported by Tian Xia revealed a stronger link between air pollution exposure and the development of respiratory diseases, including acute respiratory infections, and lung cancer, as well as cardiovascular diseases 43. Nanchong city lies in the northeastern of the Sichuan Basin, which is not conducive to the diffusion of air pollutants, dominatingly leading to the highest particulate matter concentrations 28. Therefore, controlling air pollution may be an effective measure to reduce respiratory tract infection.

## Study limitations

The limitations of our study need to be addressed: (1) elf-report information would bring recall bias. (2) Hospital-based case-control studies may bring about selection bias. (3) Another unavoidable problem is that some research volunteers couldn't accurately confirm exposures status, and categorize the duration and correlation total; the estimates were not particularly imprecise. (4) When different matching criteria are used, the matched case-control statistical results may be different. Future studies and potential analysis should consider these factors.

## Conclusion

In Nanchong city, more attention should be paid to mothers who had a history of respiratory infections, exposure to polluted water, adverse emotions during pregnancy and nutritional deficiency as these mothers may be likely to have a baby suffering from CHD. The risk model might be an effective tool for predicting of the risk of CHD in offspring by maternal experience during pregnancy, which can be used for clinical practise in Nanchong area.

## Figures and Tables

**Table 1:** Case-Control Matching Statistics

Match Type	Count
Exact Matches	93
Fuzzy Matches	234
Unmatched Including Missing Keys	40
Unmatched With Valid Keys	40
Sampling	Without Replacement
Log File	None
Maximize Matching Performance	Yes

**Table 2:** Case-Control Match Tolerances

Match Variables	Value	Fuzzy Match Attempts	Incremental Rejection Percentage
Exact (All Variables)		466.3000	98.006
Gestational Age	1.000	4570.000	90.263
Maternal Age of Pregnancy	0.000	445.000	47.416

**Table 3:** Univariate analysis of research factors among cases and controls

Research factors	Controls (N=327)		Cases (N=327)		χ^2^ value	*P* value
	N	%	N	%		
**Permanent residence**					0.646	0.421
Rural area	80	75.5	89	27.2		
Downtown	247	24.5	238	72.8		
**Ethnicity**					0.358	0.549
Han	262	80.1	268	82.0		
Minorities	65	19.9	59	18.0		
Education level*					9.100	0.011
Primary school and below	45	13.8	44	13.5		
Middle school	76	23.2	110	33.6		
College and above	206	63.0	173	52.9		
History of abnormal reproduction*					8.203	0.004
None	299	91.4	275	84.1		
Yes	28	8.6	52	15.9		
Family history of congenital heart disease*					4.098	0.043
None	290	88.7	272	83.2		
Yes	37	11.3	55	16.8		
**BMI**					4.310	0.230
Thin (≤18.4)	36a	11.0	45a	13.8		
Normal (18.5-23.9)	192a	58.7	171a	52.3		
Overweight (24.0-27.9)	90a	27.5	95a	29.1		
Obese (≥28.0)	9a	2.8	16a	4.9		
Respiratory infections*					10.910	0.001
None	267	81.7	231	70.6		
Yes	60	18.3	96	29.4		
Infection of female reproductive system*					8.724	0.003
None	318	97.2	301	92.0		
Yes	9	2.8	26	8.0		
Medication history*					8.402	0.004
None	262	80.1	230	70.3		
Yes	65	19.9	97	29.7		
Electrical radiation*					7.618	0.022
None	104	31.8	85	27.8		
Yes	223	68.2	247	72.2		
**Noise**					2.810	0.094
None	300	91.7	287	87.8		
Yes	27	8.3	40	12.2		
Heavy metals*					6.146	0.013
None	310	94.8	288	89.6		
Yes	17	5.2	34	10.4		
Pesticides*					7.181	0.007
None	318	97.2	303	92.7		
Yes	9	2.8	24	7.3		
**Organic solvents**					2.353	0.125
None	285	87.2	271	82.9		
Yes	42	12.8	56	17.1		
**Air pollution**					3.570	0.059
None	274	83.8	255	78.0		
Yes	53	16.2	72	22.0		
Water pollution*					22.731	0.000
None	305	93.3	264	80.7		
Yes	22	6.7	63	19.3		
Active smoking*					6.498	0.039
None	294	89.9	278	85.0		
Yes	33	10.1	49	15.0		
Passive smoking*					11.779	0.001
None	275	84.1	239	73.1		
Yes	52	15.9	88	26.9		
Drinking*					11.422	0.003
None	300	91.7	272	83.2		
Yes	27	8.3	55	16.8		
**Addictive drugs**					0.914	0.339
None	320	97.9	316	96.6		
Yes	7	2.1	11	3.4		
Sleep disorder*					4.407	0.036
None	310	94.8	293	90.5		
Yes	17	5.2	29	9.5		
Adverse emotions during pregnancy*					56.652	0.000
None	245	74.9	156	48.4		
Yes	82	25.1	171	52.3		
Nutrition supplementation*					14.371	0.000
None	180	55.0	227	69.4		
Yes	147	45.0	100	30.6		
Periodic prenatal examination*					4.155	0.042
None	139	42.5	165	50.5		
Yes	188	57.5	162	49.5		

Note: BMI: Body mass index; *Statistically significant after univariate analysis with a test criterion of 0.05; Each subscript letter denoted a subset of categories whose column proportions do not differ significantly from each other at the 0.05 level.

**Table 4:** Results of multivariate conditional logistic analysis

Factor	B	S.E	*P* value	OR	95% CI
Respiratory infections	0.686	0.202	0.001	1.986	1.336~2.952
Exposure to polluted water	1.176	0.280	0.000	3.242	1.872~5.613
Adverse emotions during pregnancy	1.019	0.165	0.000	2.769	2.005~3.824
Nutrition supplementation	-0.617	0.182	0.001	0.540	0.381~0.765
Constant	-2.871	0.556	0.000	0.057	

Note: B: Beta; S.E: standard error; OR: odds ratio; 95% CI: 95% confidence interval.
